# A SNP of HD-ZIP I transcription factor leads to distortion of trichome morphology in cucumber (*Cucumis sativus L.*)

**DOI:** 10.1186/s12870-021-02955-1

**Published:** 2021-04-16

**Authors:** Leyu Zhang, Duo Lv, Jian Pan, Keyan Zhang, Haifan Wen, Yue Chen, Hui Du, Huanle He, Run Cai, Junsong Pan, Gang Wang

**Affiliations:** 1 School of Agriculture and Biology, Shanghai Jiao Tong University, Shanghai, 200240 China; 2 State Key Laboratory of Vegetable Germplasm Innovation, Tianjin, 300384 China

**Keywords:** Cucumber, Trichome, Map-based cloning, Transcriptome

## Abstract

**Background:**

Trichomes are excellent model systems for the analysis of cell differentiation and play essential roles in plant protection. From cucumber inbred line ‘WD1’, we identified an EMS-induced trichome abnormally developing mutant, *nps*, which exhibited smaller, denser and no pyramid-shaped head trichomes.

**Results:**

Using F_2_ and BC_1_ populations constructed from a cross between *nps* and ‘9930’, the genetic analysis showed that the *nps* trait is controlled by a single recessive nuclear gene. We identified *CsNps* by map-based cloning with 576 individuals of the F_2_ population generated from the cross of *nps* and inbred line ‘9930’. The *CsNps* was located at a 13.4-kb genomic region on chromosome 3, which region contains three predicted genes. Sequence analysis showed that only one single nucleotide mutation (C → T) between 9930 and *nps* was found in the second exon of *Csa3G748220*, a plant-specific class I HD-Zip gene. The result of allelism test also indicated that *nps* is a novel allelic mutant of *Mict* (*Micro-trichome*). Thus, *nps* was renamed *mict-L130F*. By comparing the transcriptome of *mict-L130F* vs WD1 and 06–2 (*mict*) vs 06–1 (wildtype, near-isogenic line of 06–2), several potential target genes that may be related to trichome development were identified.

**Conclusions:**

Our results demonstrate that *Mict-L130F* is involved in the morphogenesis of trichomes. Map-based cloning of the *Mict-L130F* gene could promote the study of trichome development in cucumber.

**Supplementary Information:**

The online version contains supplementary material available at 10.1186/s12870-021-02955-1.

## Introduction

Trichomes are large specialized epidermal cells distributed on aerial parts [1–3]. In different species, the trichome can be classified as unicellular or multicellular, glandular or non-glandular, and branched or unbranched [4]. Trichomes play important roles in protecting plants from biotic and abiotic stresses, such as insect predation, excess transpiration and UV light [5, 6]. The development of unicellular and multicellular trichomes may be controlled by different pathways.

In *Arabidopsis*, as giant single epidermal cells, trichomes have become an excellent model system to study cell differentiation and development because it is easy to analyze them at the genetic, genomic and cell biology levels. Different trichome patterning methods depend on complicated gene expression and intercellular signal transduction [4]. In *Arabidopsis* leaf epidermal cells, the MYB-bHLH-WDR (MBW) complex is formed by the R2R3 type MYB transcript factor GLABRA 1 (GL1), bHLH transcript factors GLABRA 3/ENHANCER OF GLABRA 3 (GL3/EGL3), and WD40 repeat protein TRANSPARENT TESTA GLABRA 1 (TTG1). GLABRA 2 (GL2) can be activated by the MBW complex and then turn on trichome initiation [7–13]. In mature trichomes, *GL2* is highly expressed and is necessary for trichome initiation and maintenance during cell differentiation [14]. The R3 type MYB transcription factors are also activated by the MBW complex CAPRICE (CPC), ENHANCER OF TRY AND CPC 1 (ETC1), ETC2, and ETC3 which all belong to the transcription factors and act as negative regulators in trichome development [4, 15–18]. The R3 type MYB transcription factors can move to neighboring cells and interact with GL3 and TTG1, forming the different R3 type MBW complexes. This complex inhibits trichome differentiation in neighboring cells as it cannot activate *GL2* expression [18, 19].

Cucumber (*Cucumis sativus* L.) is one of the most important commercial vegetables worldwide and has become a model plant for studying the formation and development of multicellular trichomes. Trichomes are widely distributed on different organs, such as leaves, stems, branches, flowers, tendrils, and fruits, in cucumber. The trichomes on the fruits are commonly called “fruit spines”. Fruit spines directly influence fruit appearance and perceived quality. In contrast to *Arabidopsis*, a limited number of cucumber genes related to the formation and development of trichomes have been identified. Among them, *tril* (*trichome-less*) and *csgl3* (*glabrous3*) are alleles with different mutation forms of the same gene (*Csa6G514870*). In *tril* and *csgl3*, a glabrous character is observed on stems, leaves, tendrils, receptacles and ovaries, and there were no spines or tumors on the fruit surface [20–23]. The *micro-trichome* (*mict*), *tiny branched hair* (*tbh*), and *glabrous 1* (*csgl1*) alleles were identified as the result of the same 2649 bp fragment deletion in the gene *Csa3G748220*. These mutants have no noticeable trichomes on the surface of stems, leaves, tendrils, and ovaries [24–26]. The no warty phenotype of *tuberculate fruit* (*tu*) was due to complete deletion of the 4888 bp region that included the promoter and CDS region of *Csa5G577350* (*Tu*) [27].

In this study, a novel *no pyramid-shaped head trichome* (*nps*) mutant was identified in an ethyl methane sulfonate (EMS)-mutagenized WD1 (North China type) cucumber population. We isolated the *nps* gene using map-based cloning and found that *nps* is the allele of *mict* with a single nucleotide substitution. Thus, we renamed the gene *Mict-L130F*. In addition, transcriptome analysis was conducted to identify the genes involved in the formation and development of malformed trichomes/spines in cucumber. This work enriches the phenotype of trichomes and will promote the exploration of molecular mechanisms that regulate cucumber trichome development.

## Materials and methods

### Plant materials and phenotypic data collection

The *no pyramid-shaped head trichome* (*nps*) mutant was identified from an M_2_ family derived from an EMS-mutagenized cucumber WD1 (WT, North China type) population. Mutagenized plants of the first mutant generation (M_1_) were self-pollinated for two generations to make the mutated gene homozygous, and then F_2_ and BC_1_ populations were produced with the *nps* (female) and 9930 (North China type) (male) as the parents. Cucumber line 06–2 is the spontaneous *mict* mutant from the North China inbred line 06–1(wild type), which are near-isogenic lines [26]. All of the plants were grown under natural sunlight in a greenhouse at Shanghai Jiao Tong University.

The leaf phenotype of each plant was determined by visual inspection when leaves were fully expanded. The chi-square goodness of fit test was performed on phenotypic data to verify deviations from the expected 3:1 segregation in the F_2_ population or 1:1 segregation in the BC_1_ population.

### Scanning electron microscopy (SEM) analysis

Juvenile leaf (3 cm in length) and fruit (4 cm in length) samples of WT and *nps* were harvested and fixed in formaldehyde acetic acid-ethanol (FAA) which contained 50% (v/v) ethanol, 5% (v/v) acetic acid, and 3.7% (v/v) formaldehyde at 4 °C for 24 h, dehydrated through a graded ethanol series (50, 60, 70, 85, 90, 95, and 100%), critical-point dried in a Leica EM CPD030 desiccator (Leica Microsystems, Wetzlar, Germany), coated with gold-palladium in a Hitachi E-1045 ion sputter and carbon coating unit (Hitachi, Tokyo, Japan), and then observed through a JSM-6360LV scanning electron microscope (JEOL, Peabody, MA, USA) [28].

### Molecular marker development and map-based cloning

Deletion-insertion (InDel) and single nucleotide polymorphism (SNP) markers were developed based on genome resequencing. The genome of WT was resequenced on the Illumina HiSeq™ 2000 platform (Biomarker Technologies, Beijing, China). With a 30-fold sequencing depth, all of the clean reads were mapped to the ‘9930’ genome sequence (http://cucurbitgenomics.org/organism/2, version 2i). InDel and SNP sites between WD1 and 9930 were detected with the Geneious software package. Only fragments with a more than 3 bp insertion or deletion were used to develop InDel markers. For SNP genotyping, approximately 800 bp fragments, including the SNP site in the middle of the fragment, were amplified and sequenced. The primers were designed with Primer Premier 5.0. All SSR primers used in the study were kindly provided by the group of professor Sanwen Huang (Chinese Academy of Agricultural Sciences, Beijing, China).

The bulked segregant analysis (BSA) method [29] was used for screening polymorphic markers and mapping the *nps* locus. Ten individuals were randomly selected from the no pyramid-shaped head trichomes and wild type phenotype plants from the F_2_ population to create two pools (M and W DNA pools). With the 72 individuals of the F_2_ population for initial mapping, the *nps* was located on chromosome 3 (Chr3). An additional larger F_2_ population with 504 individuals generated from 9930 and *nsp* was then used for fine mapping of the *nps* locus with SNP markers and InDel markers [30–32]. Genetic maps were drawn with Join-Map 4.0. Information on all newly developed markers is provided in Table S1.

### DNA extraction and molecular marker analysis

Genomic DNA was extracted from young leaves using the CTAB method [33]. For the SSR and InDel markers, PCRs were carried out using a 10 μl volume containing 40 ng genomic DNA, 0.5 μM each primer, 200 μM dNTPs, 1× reaction buffer, and 0.5 U Taq DNA polymerase (Takara Bio Inc., Beijing, China). PCR amplification was performed on a PCR thermocycle instrument (Applied Biosystems, Foster, USA) using the following PCR program: 94 °C for 5 min; 35 cycles of 94 °C for 30 s, 50–60 °C for 30 s, 72 °C for 30 s; and a final 72 °C for 5 min. Products were separated on an 8% polyacrylamide gel by electrophoresis. After electrophoresis at 220 V for 1.5 h, the gel was separated from the plates and stained in 0.2% AgNO_3_ solution (Shanghai Shi Yi chemicals Reagent, Shanghai, China). Finally, the stained gel was transferred into the developing solution (1.5% sodium hydroxide and 0.4% formaldehyde) to reveal the silver-stained DNA bands. For SNP markers, the PCR reaction and PCR amplification were the same as for the SSR markers. The reaction samples were 20 μl in volume, and the products were analyzed by sequencing (Sangon Biotech, Shanghai, China) [34].

### Sample collection and qRT-PCR

The apical leaves (three independent biological replicates) were collected from WT and *nps*. Total RNA was extracted using an OminiPlant RNA Kit (DNase I) (CWBIO, Nanjing, China). First-strand cDNA was prepared according to the HiFiScript cDNA Synthesis Kit (CWBIO, Nanjing, China) protocol. Quantitative PCR (qPCR) was conducted using FastStart Universal SYBR Green Master Mix (ROX) (Roche) with a CFX96 Touch™ Real-Time PCR System. The cucumber *CsActin* gene was selected as an internal control [35]. Three biological replicates were used per gene. Each qRT-PCR experiment was performed with three technical replicates. The gene-specific primers are listed in Table S1.

### Materials for RNA-seq

Total RNA was extracted from the apical leaves. RNA-Seq for comparative transcriptomic analyses of the two phenotypes was performed with three biological replicates. Library construction and sequencing were performed using a BGISEQ-500 by Beijing Genomic Institution (BGI, China). The genomic DNA was removed with two digestions using amplification grade DNase I (Epigenetics, United States). The RNA was sheared and reverse transcribed using random primers to obtain cDNA, which was used for library construction. The library quality was determined using a Bioanalyzer 2100 (Agilent), and then the library was used for sequencing using the sequencing platform BGISEQ-500 (BGI, China) [36]. All the generated raw sequencing reads were filtered to remove reads with adaptors and reads in which unknown bases were greater than 10% of low-quality reads. The clean reads were obtained and saved in FASTQ format. We used Bowtie2 [37] to map the clean reads to the reference genome, (Cucumber_ChineseLong_v2 1, [38]). The read counts were summarized, and the Fragments Per Kilobase of exon per Million fragments mapped (FPKM) was calculated for each annotation on the reference sequence. The NOISeq method [39] was used to screen for DEGs between the groups. An expression trend analysis was performed using OmicShare tools (http://www.omicshare.com/tools). The clean data have been uploaded to the National Center for Biotechnology Information (NCBI) (Project ID: PRJNA706516, PRJNA706464, PRJNA706461, PRJNA706463,PRJNA706462, and PRJNA706166).

### Yeast one-hybrid assay

For the yeast one-hybrid assay, the Mict-L130F open reading frames (ORFs) were amplified from *nps* genomic DNA and then cloned into the pB42AD vector. The 2 kb promoter of CsTT4, CsFLS1, CsCER26 and CsMYB36 from WD were inserted into the vector. pB42AD-Mict-L130F and placZ-CsTT4Pro, placZ-CsFLS1Pro, placZ-CsCER26Pro, or placZ-CsMYB36Pro were co-transformed into the yeast strain EGY48a. The empty vectors were used as negative controls [40]. Primers are listed in Table S1.

## Results

### Phenotypic characterization

**Fig. 1:**
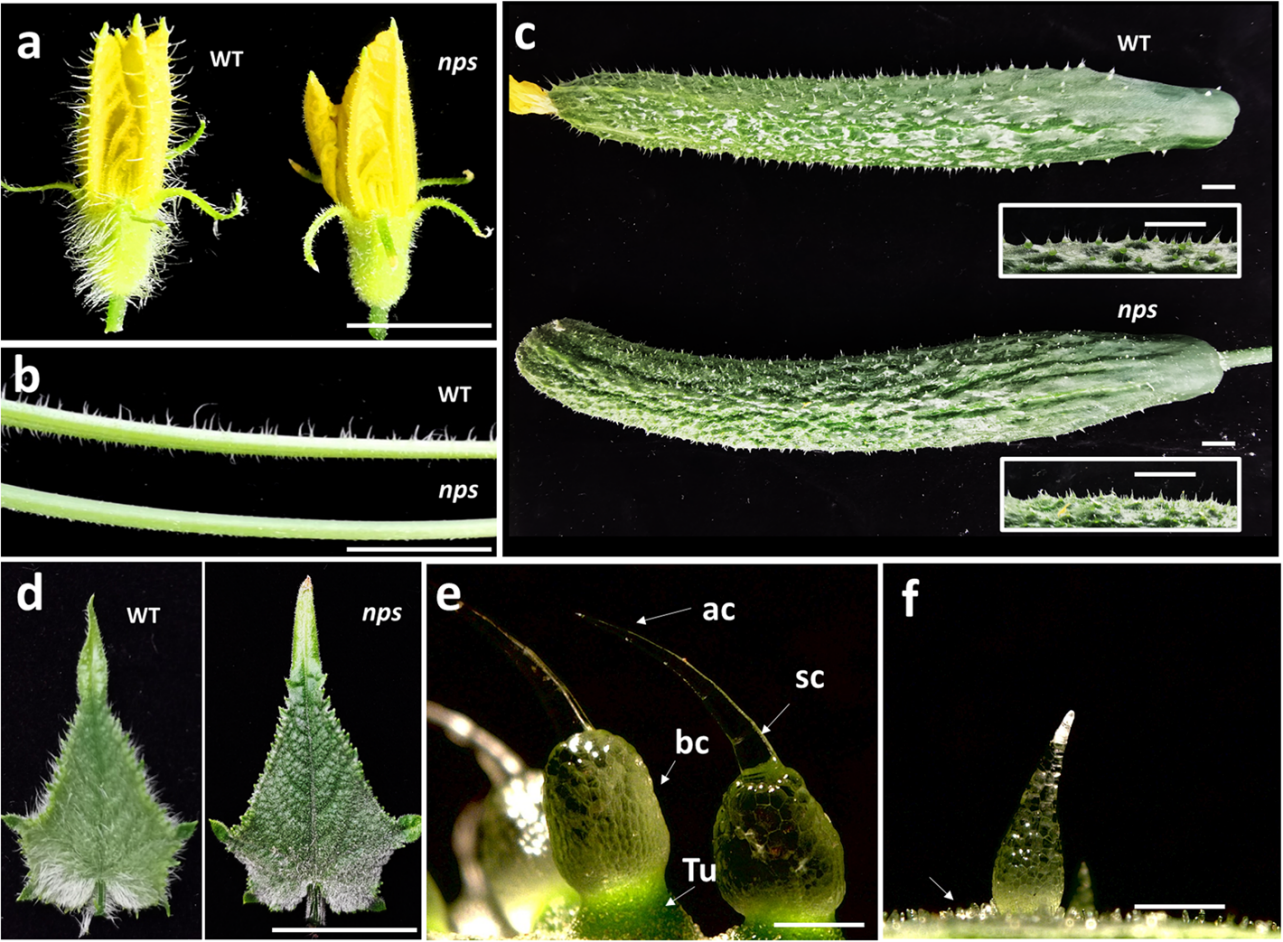
Phenotype of *nps* and WT plants. (**a**-**d**) Show male flower, tendril, fruit and leaf of WT and *nps*. **e** Fruit spines of WT; arrows indicate tubercule (Tu), base cell (bc), stalk cell (sc), and apical cell (ac). **f** Fruit spines of *nps*; arrow indicates bumps. Bars: 1 cm (a-d),100 μm (**e-f**)

Both the *nps* and WT are covered by trichomes on male flowers, tendrils, fruits and leaves. Compared with the WT trichomes, the *nps* trichomes appeared to be short and tender on leaves, female flowers, and tendrils or spines on fruit (Fig.1 a-d). 9930, which was used for producing the F_2_ population for map-based cloning, shows a similar phenotype to WT in leaves, stems, and other organs. We examined the cucumber fruit spines with an optical microscope. Obviously, spines on the WT fruits are much larger than those on the *nps* fruits. In addition, the structures of the fruit spines are different between WT and *nps*. In the WT, the spines consist of pyramid-shaped apical cells, slender and elongated stalk cells and enlarged base cells. The tuberculate connects the spines to the epidermis (Fig. 1e). In the *nps*, the fruit spines are conical in shape but without sharp tips. Many small and dense bumps are visible around the spines (Fig. 1f).

**Fig. 2:**
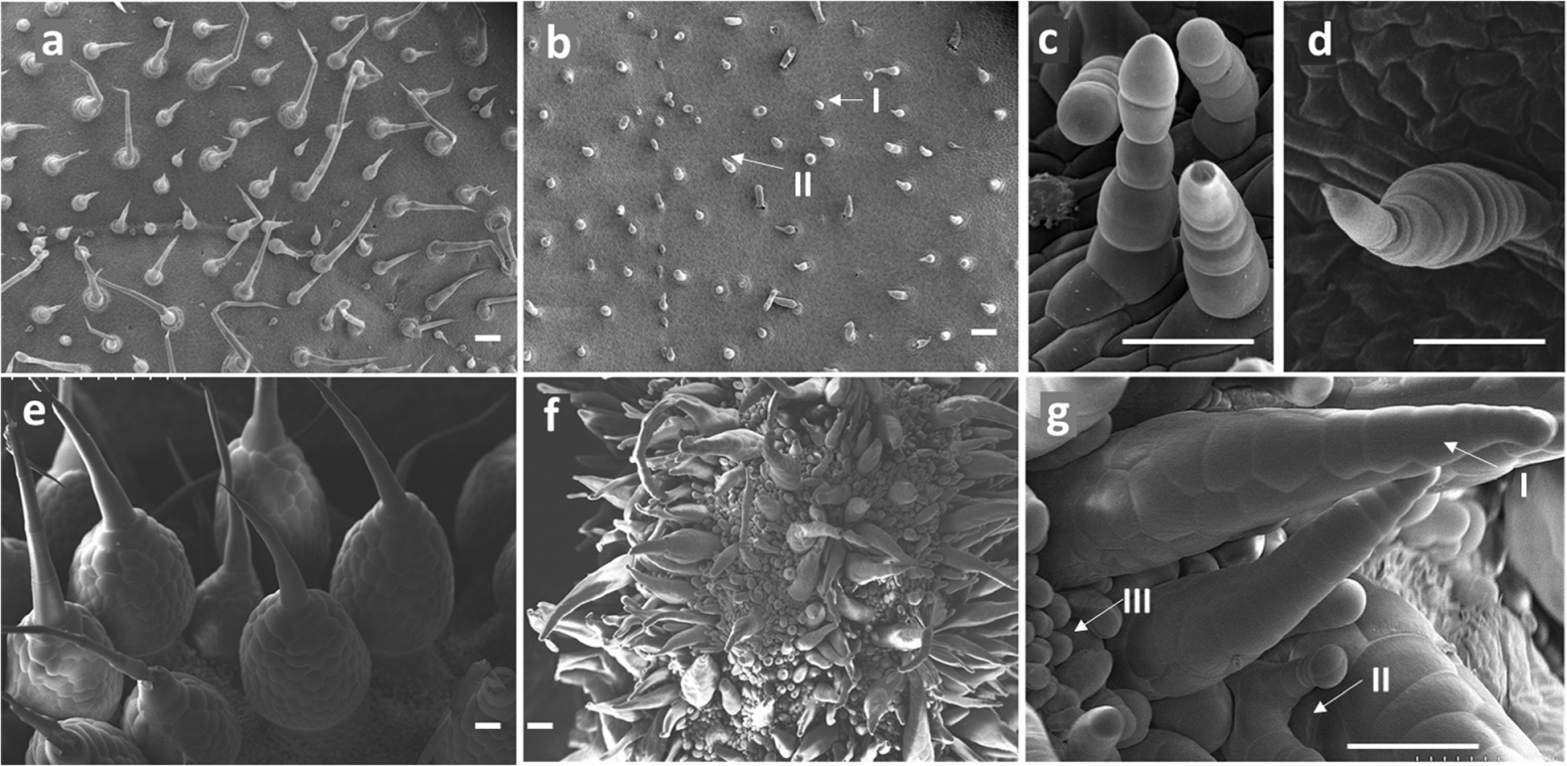
SEM images of trichomes and fruit spines between the WT and *nps*. **a** trichomes on WT leaf. **b**-**d** trichomes on *nps* leaf. **e** spines on WT fruit. (f) and **g** spines on *nps* fruit. Arrows indicate type I, type II and type III. Scale bars: 50 μm (a-d), 100 μm (**e-g**)

To characterize the morphology of cucumber trichomes in detail, we observed leaf trichomes and fruit spines with SEM. In the WT, the majority of trichomes are non-glandular and consist of pyramid-shaped apical cells, multicellular stalks with two to four elongated cylindrical-shaped cells and pie-shaped base cells. The minority were glandular trichomes and had a mini-round shape (Fig. 2a). In the *nps*, there were no enlarged basal cells in the trichome, and flat cylinder stalk cells connected the apical cell to the leaf surface (Fig. 2b). There are two types (type I and type II) of trichomes in the mutant. The main difference between type І and type II is the shape of apical cells. The apical cell shape of type I (Fig. 2c) is round; however, the shape of type II (Fig. 2d) is papillar-shaped.

In the WT, the fruit spines were distinct with clear fruit tubercules and the base of the spine was made of hundreds of spherical-shaped cells whose sizes were smaller than those of the stalk cells. The stalk consists of three to seven cylindrical-shaped cells plus a pyramid-shaped apical cell (Fig. 2e). In the *nps*, there are three types (type I, type II, and type III) of spines, which are crowded together (Fig. 2f). Type I is branchless and cone-shaped without sharp apical cells and fruit tubercules. The whole spines consist of spherical-shaped cells similar to the base of the wild-type spine with three to four flat cells on the top. Type II has branches, and the apical cell is spherical. The size of type II is smaller than that of type I because type ii has fewer cells. Type III is the smallest, with a greatly reduced number of cells and it is difficult to separate the base and stalk; however, the apical cell is also spherical (Fig. 2g).

**Fig. 3:**
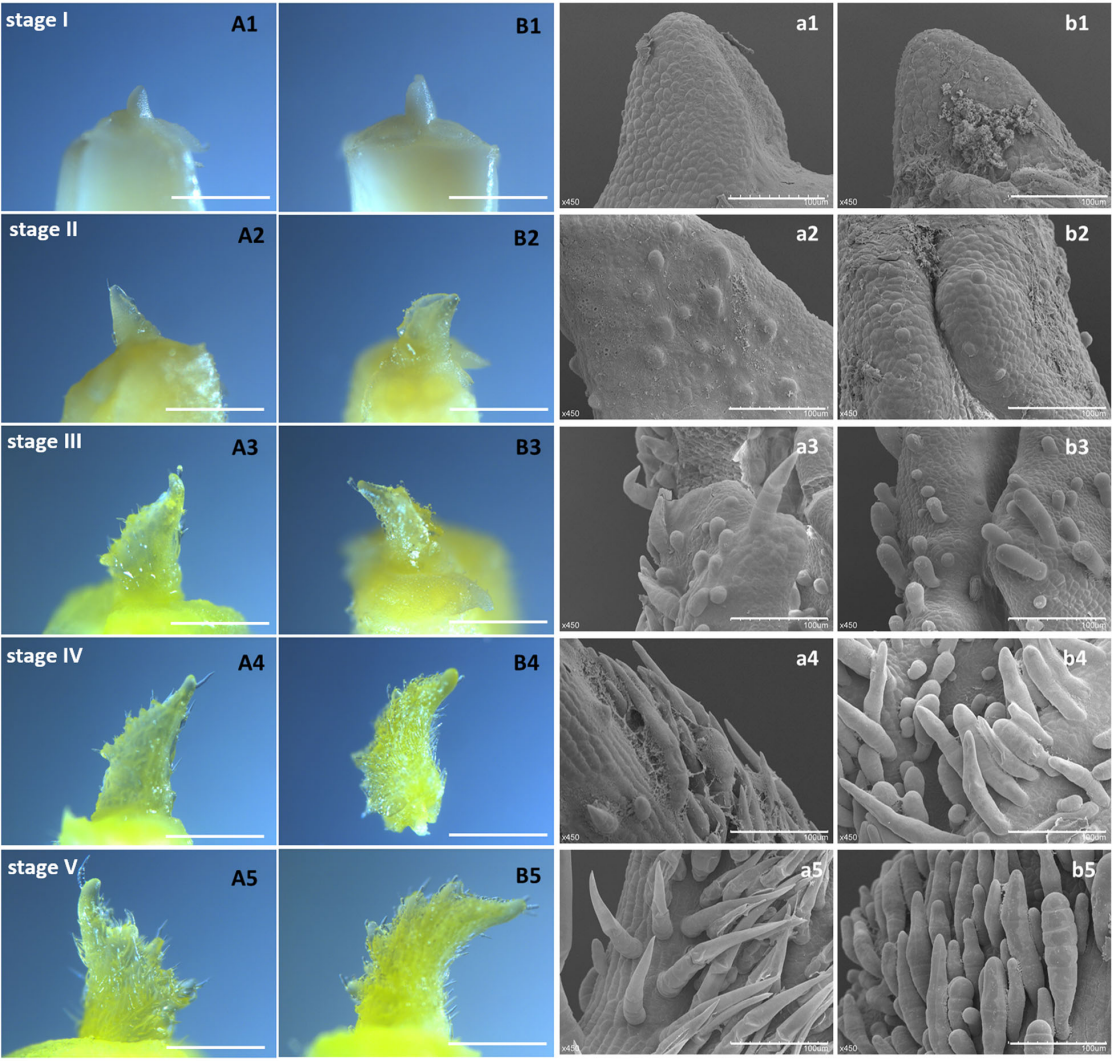
Five stages of trichomes morphological development. (A1-A5) and (a1-a5) are leaves of WT. (B1-B5) and (b1-b5) are leaves of *nps*. Bars: 1 cm (A1-A5, B1-B5),100 μm (a1-a5, b1-b5)

To further detect the development of trichomes, we observed changes in the leaf primordium during germination between the WT and mutant through optical microscopy and SEM. Based on the morphology, the initiative development of trichomes is divided into five stages (Fig. 3). In stage I, no trichomes appear on the leaf primordium. In Stage II, several bulges emerge on the leaf surface. In Stage III, a mass of bulges form, and their shapes are transformed into cone-like structures. In Stage IV, multicellular trichomes begin to develop. In Stage V, the morphology and density of the trichomes are basically formed and the trichomes further elongate during the development of leaves. The greatest difference between WT and *nps* occurs from Stages III to V. From Stage III, pyramid-shaped apical cells fail to develop in the *nps*. These results indicated that the *nps* functioned in the morphogenesis of trichomes.

### The *nps* locus is controlled by a recessive nuclear gene in cucumber

The trichomes/fruit spines of all the F_1_ plants produced from the cross of *nps* and 9930 were consistent with those of 9930. No significant phenotypic difference was found between homozygous dominant (*Nps/Nps*) and heterozygous (*Nps/nps*) plants. Of the 178 *nps* × 9930 F_2_ plants, 132 and 46 plants exhibited 9930 and mutant phonotypes, respectively. Goodness-of-fit χ^2^ tests indicated that the segregation ratios in the F_2_ (χ^2^ = 0.029 < χ^2^_0.05,1_ = 3.84) populations were consistent with the expected 3:1 segregation ratio. Among 186 (*nps* × 9930) × *nps* BC_1_ individuals, 87 and 99 plants exhibited 9930 and mutant phonotypes, respectively, conforming to the 1:1 segregation ratio (χ^2^ = 0.65 < χ^2^_0.05,1_ = 3.84). These results indicated that both the F_2_ and BC_1_ populations conformed to Mendel segregation which confirmed that *nps* was conferred by a single recessive nuclear gene in cucumber.

### Primary mapping of the *nps* locus

**Fig. 4:**
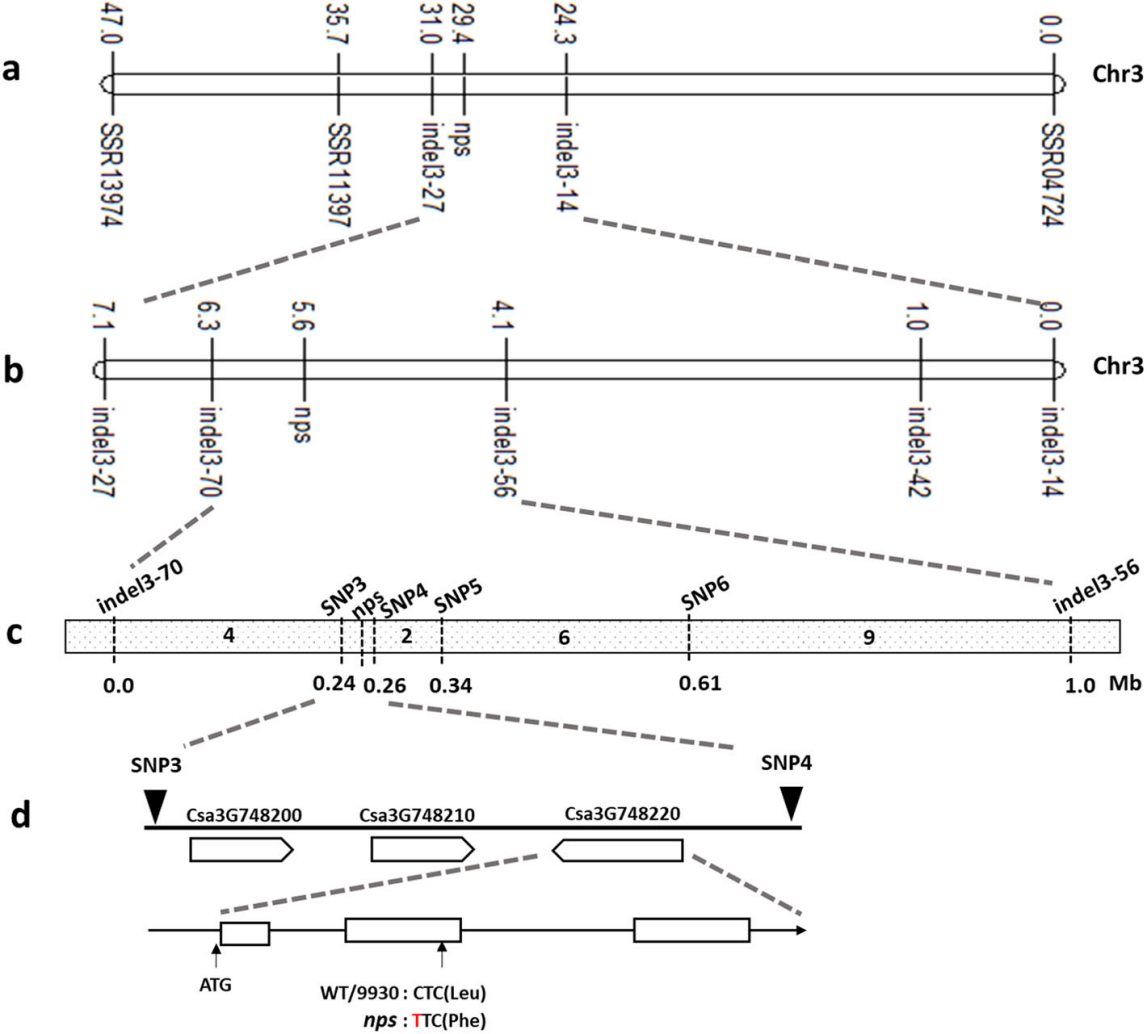
Map-based cloning of *nps* locus in cucumber. (a) The *nps* locus was located on chromosome 3. (b) The *nps* locus was further mapped to the region between Indel3–70 and Indel3–56. (c) The *nps* locus was finally narrowed to 13.4 kb between markers SNP3 and SNP4. The relative physical distances of six markers are indicated below the map based on their sequence position in the cucumber genome. The number on the chromosome represents the quantity of recombinants for the corresponding marker. (d) Three genes were predicted in the 13.4-kb region and the structure of the *nps* candidate gene *Csa3G748220*, which has three exons and a mutation site (C to T) in the second exon

Genetic mapping of the *nps* locus was performed using the BSA approach. Polymorphic SSR markers between *nps* and 9930 were identified. Based on the resequencing genomic data, 73 polymorphic InDel markers between *nps* and 9930 were developed. All 56 polymorphic SSR markers and 73 polymorphic InDel markers between *nps* and 9930 were used to analyze the W and M DNA pools. Among these markers, five markers (SSR04724, Indel3–14, Indel3–27, SSR11397, SSR13974) were polymorphic between the W and M DNA pools. The five markers were located on chromosome 3 in cucumber. Genetic linkage analysis using 72 individuals of the F_2_ population showed that *nps* was linked with these five markers (Fig. 4a). The results indicated that *nps* was located on chromosome 3, within an approximately 6.7 cM interval between Indel3–14 and Indel3–27 (Fig. 4a). To narrow down the target region, three new polymorphic InDel markers (Indel3–42, Indel3–56, Indel3–70) between Indel3–14 and Indel3–27 were developed based on the genomic sequence (Fig. 4b). The *nps* locus was mapped between the markers Indel3–56 and Indel3–70 on chromosome 3 by genotyping 504 F_2_ individuals (Fig. 4b). A total of 21 recombinants were identified between Indel 3–56 and Indel 3–70 by genotyping 504 individuals.

### Fine mapping of the *nps* locus

According to the resequencing genome data, there were no more polymorphic InDel markers between Indel3–56 and Indel3–70. Therefore, SNP markers were developed for fine mapping. Five SNP markers were developed and these SNP markers showed polymorphisms between *nps* and 9930. The 21 recombinants between Indel3–56 and Indel3–70 were used for fine mapping with the polymorphic SNP markers. Finally, the *nps* locus was mapped to a region between SNP3 and SNP4 markers, encompassing a physical distance of 13.4 kb (Fig. 4c).

### Candidate gene identification

In the 13.4-kb genomic region, three putative genes were identified in the “9930” genome database (http://cucurbit-genomics.org/organism/2). The information and predicted functions of the three genes are presented in Table 1. After analyzing the coding sequences and the protomer regions of the three genes of 9930 and *nps*, only one nonsynonymous single nucleotide mutation was identified in gene *Csa3G748220*, whereas no difference was found in the other two genes (Fig. 4d). In addition, the single nucleotide mutation of the candidate gene only existed in the *nps* mutant but not in the other 10 cucumber lines of the natural populations (File S1). The data suggested that this SNP may be the causal SNP for the trichome phenotype. We also examined the linkage relationship of this SNP with the *nps* locus. A dCAPS marker (dcaps-M) developed based on this SNP (primer information in Table S1) was used to genotype 504 *nps* × 9930 F_2_ plants. The results showed that dcaps-M co-segregated with the *nps* locus in this population (Figure S1). Therefore, we concluded that *Csa3G748220*, a class I Homeodomain-leucine zipper (HD-ZIP) gene, carrying the SNP site was the most likely candidate gene for *nps*.

**Table 1:** Predicted genes in the 13.4-kb genomic region of cucumber Chr3

Gene ID	BLASTX plant proteins	E-value	Annotation
Csa3G748200	AT2G36640	3.1e-75	Embryonic cell protein 63
Csa3G748210	AT2G36620	7.1e-76	60S ribosomal protein L24
Csa3G748220	AT5G03790	9.8e-42	Homeobox-leucine zipper protein

The genomic sequence of *Csa3G748220* from 9930 and *nps* was cloned, and the full length of *Csa3G748220* was 2930 bp in the 9930 reference genome. Sequence alignment between 9930 and *nps* indicated that a single nucleotide mutation (C → T) occurred in the second exon of the *Csa3G748220* gene, which resulted in an amino acid change from Leucine to Phenylalanine. A previous study confirmed that the recessive mutant *mict* had a 2649-bp genomic deletion in *Csa3G748220* and appeared to be glabrous with no noticeable trichomes on leaves, stems, branches and flowers, or spines on fruit [26]. Allelism test was also performed. Crossing *nps* and *mict* mutants, the F_1_ plants exhibited the micro-trichome phenotype of *mict*. Among 132 *nps* × *mict* F_2_ individuals, 33 and 99 plants exhibited no pyramid-shaped head trichomes and micro-trichome phenotypes, respectively, conforming to the 1:3 segregation ratio. These results indicated that the mutation sites of the two mutants occurred in the same gene (Figure S2), and the *nps* is an allelic mutation of *mict*, renamed as *mict-L130F.*

### Transcriptome profile analysis

To investigate the gene regulatory networks of *Mict*, comparative transcriptomic analysis of cucumber apical leaves was performed between 06 and 2 (*mict*) compared with its wild type 06–1 and *nps* (*mict-L130F*) compared with its wild type WD1. The transcriptomic data of 06–2 and 06–1 is from previous study [40] and in this study the transcriptome of *nps* (*mict-L130F*) and WD1 was measured. We generated 49.46–51.38 million raw reads from each library, and 45.69–47.62 million clean reads were obtained after the removal of low-quality reads and adapter sequences. Among the clean reads, 93.96–96.24% were mapped to the gene database. Gene expression levels were calculated by FPKM values and differential expression was defined by statistical parameters (*P* < 0.05 and fold change > 2 or < −2).

**Fig. 5:**
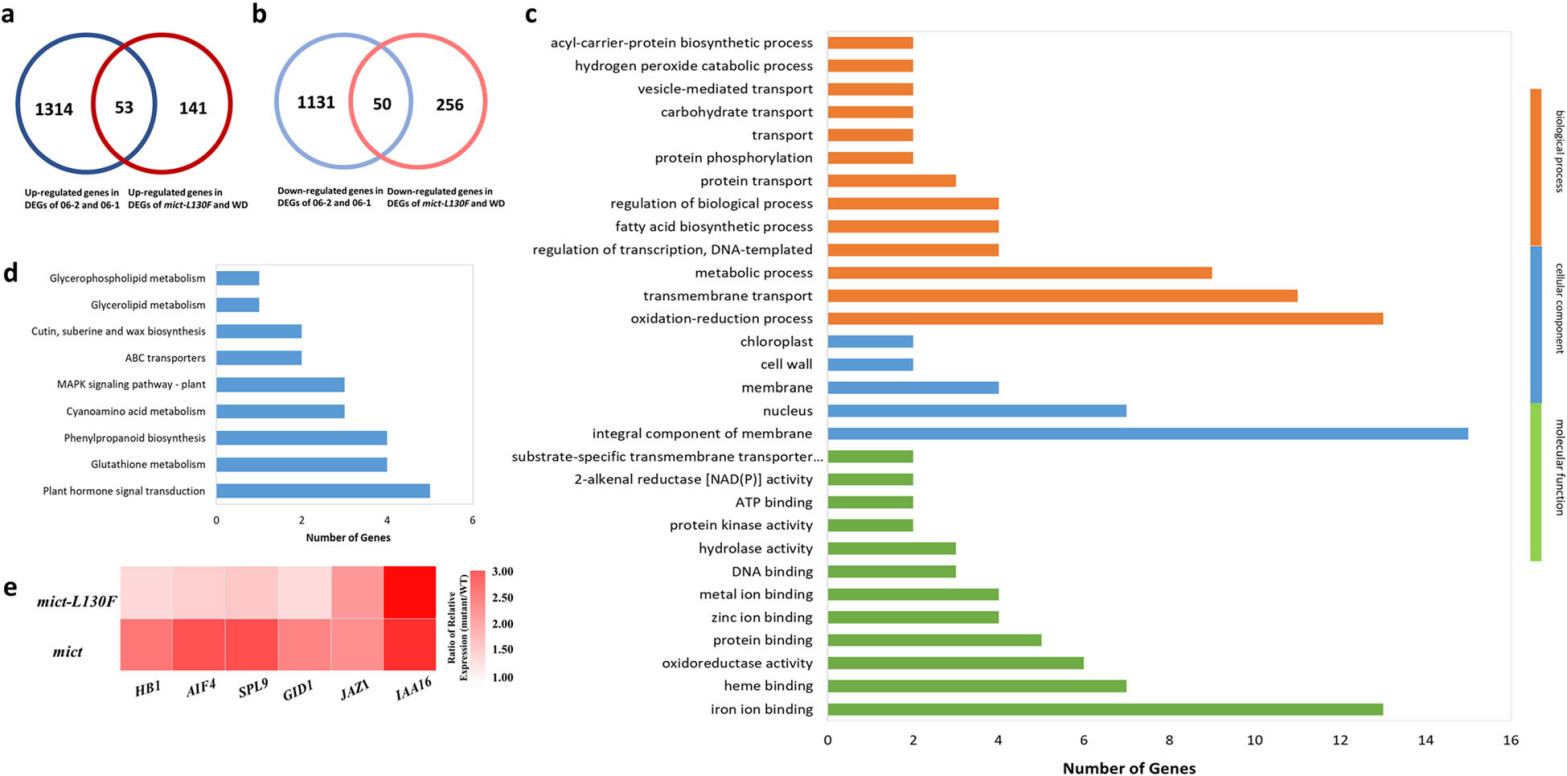
Analysis of different expression genes between *mict-L130F* vs WD1 and 06–2 vs 06–1. (a) Venn diagrams of up-regulated genes in DEGs of *mict-L130F* VS WD1 (red) and 06–2 VS 06–1(blue) (b) Venn diagrams of down-regulated genes in DEGs of *mict-L130F* VS WD1 (red) and 06–2 VS 06–1(blue) (c) Go terms were categorized into biological process, cellular components and molecular functions based on the number of genes. (d) KEGG pathways that were enriched in the differentially expressed genes. (e) Expression of the candidate genes involved in the trichome development

At a false discovery rate (FDR) of 0.05, the group of *mict-L130F* vs WD1 was analyzed based on the RNA-Seq data of three independent biological replicates. A total of 500 differentially expressed genes (DEGs) were identified in *mict-L130F* vs WD1, including 194 up-regulated and 306 down-regulated genes (Table S2). Further analysis found that 53 genes and 50 genes were up-regulated or down-regulated respectively, in both groups, *mict-L130F* vs WD1 and 06–2 vs 06–1 (Fig. 5a, b). Therefore, the 103 DEGs were thought to be related to trichome development in cucumber. To determine the functions of these DEGs, gene ontology (GO) term enrichment and KEGG analysis were performed. The most significantly enriched GO terms were ‘oxidation-reduction process’ in biological process, ‘integral component of membrane’ in cellular component and ‘iron ion binding’ in molecular function (Fig. 5c). Based on the KEGG database, pathway enrichment analysis was performed to identify significantly enriched plant hormone signal transduction, Glutathione metabolism, phenylpropanoid biosynthesis, Cyanoamino acid metabolism and MAPK signaling pathways (Fig. 5d).

Furthermore, we categorized the putative functions of these DEGs and focused on six genes which were involved in plant hormone pathway or encode critical transcription factors that can be linked to trichome formation based on work in other systems (Table 2). To further confirm the DEGs identified by RNA-Seq analysis, we tested the transcriptomic data by checking the expression of the genes shown in Table 2 with quantitative reverse transcription polymerase chain reaction (qRT-PCR) and found a good agreement with the transcriptome data (Figure S3). Except the *JAZ1-like* gene and *IAA16-like* gene, the relative expression fold change of *mict-L130F*/WD1 was higher than that of 06–2/06–1 in other genes (Fig. 5e). In Table 2, three genes encoding critical transcription factors can be linked to trichome development. The putative homolog of *Csa1G051590* in *Arabidopsis thaliana* is an *SPL9-like* gene that promotes trichome formation in the adult phase by regulating miR172 and its target of *Eat 1* (*TOE1*) and *TOE2* [40, 41]. The putative homolog of *Csa5G604260* in *Arabidopsis thaliana* encodes a HD-Zip I transcriptional activator involved in leaf and hypocotyl development. Its promoter is bound by PIF1 which likely regulates its expression [42]; The putative homolog of *Csa1G555600* in *Arabidopsis thaliana* negatively regulates cell elongation through a triantagonistic bHLH system [43].

**Table 2:** Candidate genes involved in trichome formation of cucumber

Gene ID	*Arabidopsis* homolog	Annotation
Csa1G051590	AT2G42200.1	Squamosa promoter binding protein SPL9-like
Csa5G604260	AT3G01470.1	Homeobox-leucine zipper-like protein HB1-like
Csa1G555600	AT1G09250.1	Transcription factor AIF4-like
Csa1G397130	AT3G04730.1	Auxin-responsive protein IAA16-like
Csa7G391240	AT3G63010.1	Gibberellin receptor GID1-like
Csa1G597690	AT1G19180.1	Jasmonate-zim-domain protein 1 JAZ1-like

**Fig. 6:**
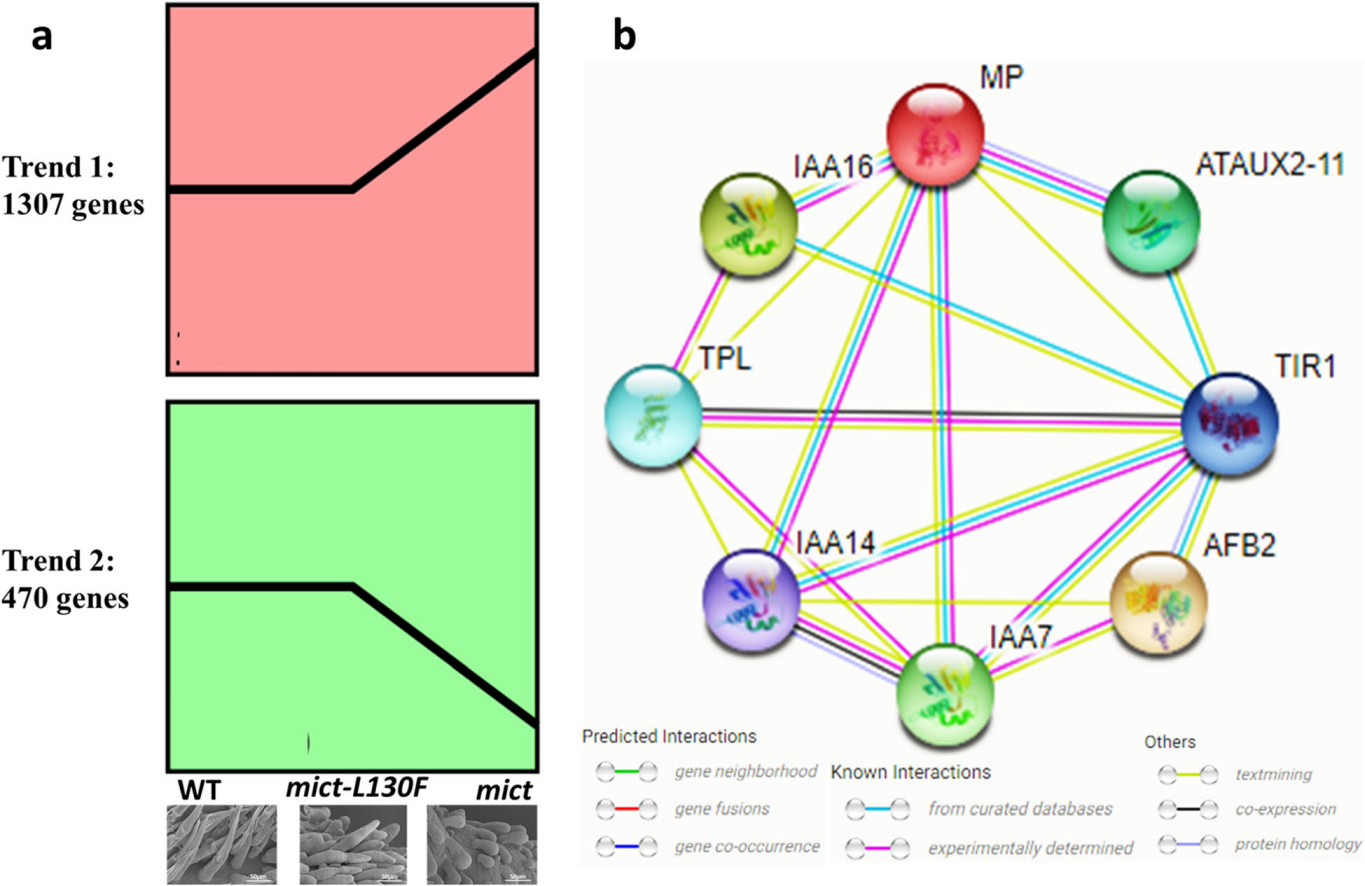
Transcriptome analysis of 06–2(*mict*) vs *mict-L130F*. (a) Expression trend analysis of DEGs (b) Homologs that were associated with auxin respond

In case to find out genes function on the different trichomes morphology, especially trichome elongation, we performed transcriptome analysis of 06–2 vs *mict-L130F*. A total of 2184 DEGs was identified. Among them, 1706 genes were down-regulated and 478 genes were up-regulated in *mict-L130F*. Expression trend analysis of 2184 DEGs was performed and nearly 81.3% DEGs were classified into 2 trends (Fig. 6a). SEM shows that the leaf trichomes length of WT and *mict-L130F* was comparatively similar and that of *mict* was significantly shorter. Trend 2 is similar to the trend of trichome length and Trend 1 is opposite, which means these genes may function on the length of trichomes. Phytohormone treatments influence cucumber trichome development and morphogenesis [44–46]. STRING analysis [47] was used to predict the functional association networks of the homologs to Arabidopsis among these DEGs. In the predicted protein interaction network, functionally related genes that were closely associated with auxin response were identified (Fig. 6b), which provide targets for further studies of trichome development.

## Discussion

Cucumber fruits are economically valuable and fruit spines directly affect the appearance and perceived quality of the fruits. No pyramid-shaped head mutant (*nps*) used in this article was selected from the M_2_ generation of the WD1 mutant library, which was caused by EMS mutagenesis. Genetic studies indicated that the no pyramid-shaped head trait is recessive to the pyramid-shaped head trait and the *mict-L130F* mutant in *Mict* gene induced a change in amino acid from leucine (L) to phenylalanine (F). In a previous study, the determination and morphogenesis of the multicellular trichomes of cucumber were regulated by HD-ZIP transcription factors. The genes related to cucumber trichome (spine) development are *tril*, *mict* and *Tu* [20, 26, 27]. In this study, *mict-L130F* showed an intermediate trichome phenotype between *mict* and wild type. The abnormal trichomes/spines are all without pyramid-shaped heads and appear to be softer than those of the wild type, which is different from known types of trichomes.

The development of glandular trichomes has multiple stages, including initiation and expansion of the trichome precursor cell protuberating out of the epidermal surface, followed by periclinal bipartition to two cells (top and bottom) which later form the head region and the stalk, respectively, through subsequent cell divisions [45]. Disruption of the *Mict* locus led to abnormal trichomes with a greatly reduced number of cells, aberrant cell shapes and organization, and branched trichomes (Fig. 1), suggesting that the *Mict* gene may be required for cell division and directional growth of cucumber trichomes. By observing the *mict-L130F* and WT leaves continuously, we found that the formation of apical cells was prevented in stage III, but the elongation of trichomes was not affected in stage IV and stage V in *mict-L130F*. In previous study, we observed *mict* leaves in the same method and found that trichomes stayed in the bulge shape (stage III) without cell division or elongation [48]. In addition, the density of trichomes/spines in *mict* or *mict-L130F* are also higher than those of WT (Fig. 2, [26] These results implied that *Mict* functioned at the beginning of trichome development, particularly in trichome initiation from single-cell bulges to cone-shaped multicellular trichomes.

According to allele verification, F_1_ progeny are micro-trichomes and F_2_ progeny shows a ratio of 3:1 which confirm that *mict* and *mict-L130F* mutants are caused by allelic mutations of the same gene *Csa3G748220.* Different with *mict-L130F* mutants, in F_1_ progeny, there only single copy of *mict-L130F* and the expression level of the gene may be affected. It has been reported that *Tril* has a gene dosage effect on fruit spine density [35]. Thus, we speculated that whether there is also a gene dosage effect existing in *Mict* and further research are needed.

Based on our previous study, *mict* mutant leaves and fruit surfaces looked brighter and glossier and it has been confirmed that Mict can activate four genes (*CsFLS1*, *CsTT4*, *CsCER26* and *CsMYB36*) involved in flavonoid and cuticular lipids biosynthesis which related to the appearance [48]. In this study, we also found that *mict-L130F* shows the similar phenotype with *mict* in wax and glossy (Fig. 1). Transcriptome profiling reveals that the four genes are all down-regulated both in *mict* and *mict-L130F*, and the expression levels of these genes were more affected in *mict* mutants. Consistent with Mict, in yeast one-hybrid assay, Mict-L130F can also activate these four genes (Fig. S4). It declares that the single amino acid mutant of Mict may also affect the regulation of secondary metabolites.

Plant trichome initiation and morphogenesis are influenced by diverse developmental and environmental cues [49–51]. KEGG analysis of these DEGs revealed that plant hormone signal transduction was affected in *Mict-L130F* and *mict* (Fig. 5d). The DEGs involved in the hormone pathway may be related to trichome formation, as listed in Table 2, and they were all up-regulated in both *mict* and *Mict-L130F* (Fig. 5e). Csa1G397130 is an AUX/IAA transcriptional regulator family protein. Aux/IAAs proteins and auxin response factors (ARFs) are involved in auxin-dependent transcriptional regulation, and ARFs can act as either transcriptional activators or repressors of auxin-responsive genes [52, 53]. Down-regulation of SlIAA15 in tomato obviously decreased the formation of different types of trichomes, which indicates that auxin-dependent transcriptional regulation is involved in trichome initiation in tomato [54, 55]. The putative homolog of Csa7G391240 in *Arabidopsis thaliana* encodes the gibberellin receptor. Gibberellin (GA) hormones are involved in many growth and developmental processes in plants. GA-deficient mutants, such as *ga1–3*, have almost completely glabrous leaves. The application of exogenous GAs to *ga1–3* plants induces trichome formation [56]. Gibberellins promote trichome production in *Arabidopsis* through up-regulation of *GL1* and *TTG* genes [57]. In cucumber, the application of GA showed a positive effect on fruit trichome initiation [45]. *CsTTG1* acts in parallel to *Mict*/*csgl1*, a key trichome formation factor that regulates the initiation of fruit trichome and *CsTTG1* can directly interact with *Mict* [58]. Whether the functions of GA on fruit spines are related to *GL1* and *TTG* remains to be further studied. Csa1G597690 is a jasmonate-zim-domain (JAZ-like) protein that acts as a transcriptional repressor in the jasmonate (JA) hormonal response. The hormonal signals GA and JA antagonistically and synergistically regulate diverse aspects of plant growth, development, and defense. JA activates the WD-repeat/bHLH/MYB complex and induces initiation of trichomes in *Arabidopsis* [59]. In cucumber, trichomes are multicellular, unbranched cells that are regularly distributed on most aerial surfaces. The *tril/csgl3* and *csgl1/mict* are important cucumber mutants that enable the dissection of trichome development into distinct, genetically controlled steps, as follows: *Tril* regulates the initiation and density of the trichomes and *Mict* allows trichomes to differentiate correctly [20, 26]. Both *Tril* and *Mict* are HD-ZIP transcription factors, different from the well-known MYB-WD40-bHLH complex in *Arabidopsis*. The regulatory network for the formation of trichomes remains unknown. However, *Tril* and *Mict* may also be involved in the JA pathway and need more experimental data.

## Conclusions

Through morphological observation, we found that several types of trichomes mutant forms existed in the *no pyramid-shaped head trichomes* mutant. Map-based cloning confirmed that the mutant was caused by the single amino acid substitution of the gene *Csa3G748220*, which is an allelic mutant of *Mict.* According to the transcriptome analysis of *mict-L130F* and *mict*, we identified that several genes may be related to the phenotype differences. Our results can help further facilitate understanding of the biological and molecular mechanisms of trichomes development.

## Supplementary Information

**Additional file 1 Figure S1** Partical results of linkage analysis with dcaps-M demonstrating that this marker is co-segregated with the phenotype.

**Additional file 2 Figure S2** Phenotype of leaves on *nps*, *mict* and F_1_(*nps*×*mict*).

**Additional file 3 Figure S3** qRT-PCR confirmation of differentially expressed genes identified by transcriptome analysis.

**Additional file 4 Figure S4** Mict-L130F activates the expression of *CsTT4*, *CsFLS1*, *CsCER26*, and *CsMYB36*.

**Additional file 5 File S1** Amino acid sequence alignment of Mict between 10 cucumber natural lines and mutant *nps*.

**Additional file 6 Table S1** List of primers used in this study.

**Additional file 7 Table S2** List of genes that are differentially expressed in the wild-type and *mict-L130F* mutant.

**Additional file 8 Table S3** List of genes that are consistently differentially expressed in both groups, mict-L130F vs WD1 and 06–2 vs 06–1.

**Additional file 9 Table S4** List of genes that are consistently differentially expressed in 06–2 vs mict-L130F.

## Data Availability

All data generated or analyzed during this study are included in this published article [and its additional files].

## Abbreviations

NPS: No pyramid-shaped head trichomes
EMS: Ethyl methane sulfonate
BSA: Bulked segregation analysis
InDel: Insertion deletion
SNP: Single nucleotide polymorphism
SSR: Simple Sequence Repeats
WT: Wild type
HD-ZIP: Homeodomain-leucine zipper
dCAPS: derived cleaved amplified polymorphic sequences.
SEM: Scanning electron microscope
KEGG: Kyoto Encyclopedia of Genes and Genomes
GO: Gene Ontology
DEGs: Different Expression Genes
