# Supplementary material for: A SNP of HD-ZIP I transcription factor leads to distortion of trichome morphology in cucumber (Cucumis sativus L.)
Source: BMC Plant Biol. 2021 Apr 16;21:182. doi: 10.1186/s12870-021-02955-1 (PMC8052656; doi:10.1186/s12870-021-02955-1)
Supplement: Supplementary file 3 — Additional file 3 Figure S3 qRT-PCR confirmation of differentially expressed genes identified by transcriptome analysis. [file 12870_2021_2955_MOESM3_ESM.pdf]

A SNP of HD-ZIP I transcription factor leads to distortion of trichome morphology in cucumber (*Cucumis sativus L.*)

Leyu Zhang[1], Duo Lv[1], Jian Pan[1], Keyan Zhang[1], Haifan Wen[1], Yue Chen[1], Hui Du[1], Huanle He[1], Run Cai[1,2], Junsong Pan[1*], Gang Wang[1*]

[1]School of Agriculture and Biology, Shanghai Jiao Tong University, Shanghai 200240, China.

[2]State Key Laboratory of Vegetable Germplasm Innovation, Tianjin 300384, China.

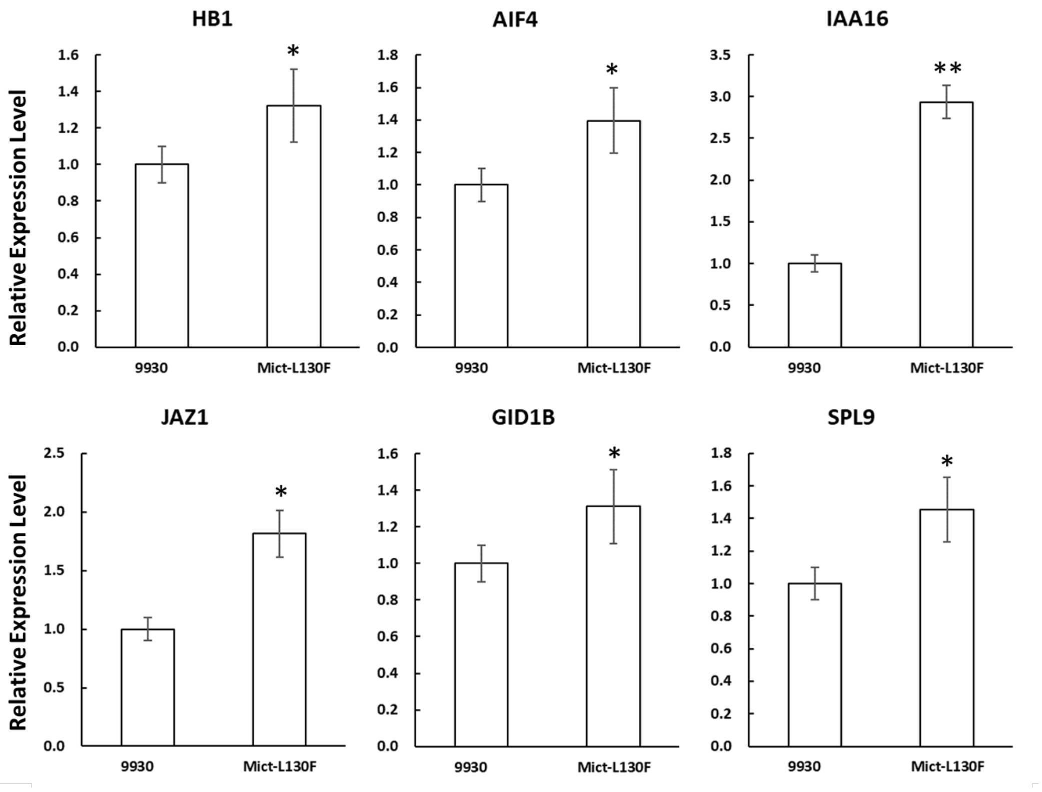

Figure S3. qRT-PCR confirmation of differentially expressed genes identified by transcriptome analysis. The relative expression of the 6 candidate genes validated by qRT-PCR in *mict-L130F* and 9930 leaves. Error bars indicate SD (n=4). Student's t-test: *, P<0.05; **, P <0.01.
