# Supplementary material for: A SNP of HD-ZIP I transcription factor leads to distortion of trichome morphology in cucumber (Cucumis sativus L.)
Source: BMC Plant Biol. 2021 Apr 16;21:182. doi: 10.1186/s12870-021-02955-1 (PMC8052656; doi:10.1186/s12870-021-02955-1)
Supplement: Supplementary file 5 — Additional file 5 File S1 Amino acid sequence alignment of Mict between 10 cucumber natural lines and mutant nps. [file 12870_2021_2955_MOESM5_ESM.pdf]

A SNP of HD-ZIP I transcription factor leads to distortion of trichome morphology in cucumber (*Cucumis sativus L.*)

Leyu Zhang[1], Duo Lv[1], Jian Pan[1], Keyan Zhang[1], Haifan Wen[1], Yue Chen[1], Hui Du[1], Huanle He[1], Run Cai[1,2], Junsong Pan[1*], Gang Wang[1*]

[1]School of Agriculture and Biology, Shanghai Jiao Tong University, Shanghai 200240, China.

[2]State Key Laboratory of Vegetable Germplasm Innovation, Tianjin 300384, China.

File S1

Amino acid sequence alignment of Mict between 10 cucumber natural lines and mutant *nps*. The amino acid mutation occured at positions 130 from L to F in *nps*, which are highlighted in yellow.

```
CLUSTAL O(1.2.4) multiple sequence alignment
9930 MEWATGSFRPFVSRAPESSFGFLYNYNLEQFQGVDVKHSAMAGASETVLQGLVPGMDMNS 60
WD   MEWATGSFRPFVSRAPESSFGFLYNYNLEQFQGVDVKHSAMAGASETVLQGLVPGMDMNS 60
GL3  MEWATGSFRPFVSRAPESSFGFLYNYNLEQFQGVDVKHSAMAGASETVLQGLVPGMDMNS 60
TMG1 MEWATGSFRPFVSRAPESSFGFLYNYNLEQFQGVDVKHSAMAGASETVLQGLVPGMDMNS 60
7088 MEWATGSFRPFVSRAPESSFGFLYNYNLEQFQGVDVKHSAMAGASETVLQGLVPGMDMNS 60
g38  MEWATGSFRPFVSRAPESSFGFLYNYNLEQFQGVDVKHSAMAGASETVLQGLVPGMDMNS 60
06-1 MEWATGSFRPFVSRAPESSFGFLYNYNLEQFQGVDVKHSAMAGASETVLQGLVPGMDMNS 60
422  MEWATGSFRPFVSRAPESSFGFLYNYNLEQFQGVDVKHSAMAGASETVLQGLVPGMDMNS 60
S05  MEWATGSFRPFVSRAPESSFGFLYNYNLEQFQGVDVKHSAMAGASETVLQGLVPGMDMNS 60
S06  MEWATGSFRPFVSRAPESSFGFLYNYNLEQFQGVDVKHSAMAGASETVLQGLVPGMDMNS 60
nps  MEWATGSFRPFVSRAPESSFGFLYNYNLEQFQGVDVKHSAMAGASETVLQGLVPGMDMNS 60
     ************************************************************
9930 YGNLEKKKRLSSEQLESLERSFQEEIKLDPDRKQKLSKELGLQPRQIAVWFQNRRARWKA 120
WD   YGNLEKKKRLSSEQLESLERSFQEEIKLDPDRKQKLSKELGLQPRQIAVWFQNRRARWKA 120
GL3  YGNLEKKKRLSSEQLESLERSFQEEIKLDPDRKQKLSKELGLQPRQIAVWFQNRRARWKA 120
TMG1 YGNLEKKKRLSSEQLESLERSFQEEIKLDPDRKQKLSKELGLQPRQIAVWFQNRRARWKA 120
7088 YGNLEKKKRLSSEQLESLERSFQEEIKLDPDRKQKLSKELGLQPRQIAVWFQNRRARWKA 120
g38  YGNLEKKKRLSSEQLESLERSFQEEIKLDPDRKQKLSKELGLQPRQIAVWFQNRRARWKA 120
06-1 YGNLEKKKRLSSEQLESLERSFQEEIKLDPDRKQKLSKELGLQPRQIAVWFQNRRARWKA 120
422  YGNLEKKKRLSSEQLESLERSFQEEIKLDPDRKQKLSKELGLQPRQIAVWFQNRRARWKA 120
S05  YGNLEKKKRLSSEQLESLERSFQEEIKLDPDRKQKLSKELGLQPRQIAVWFQNRRARWKA 120
S06  YGNLEKKKRLSSEQLESLERSFQEEIKLDPDRKQKLSKELGLQPRQIAVWFQNRRARWKA 120
nps  YGNLEKKKRLSSEQLESLERSFQEEIKLDPDRKQKLSKELGLQPRQIAVWFQNRRARWKA 120
     ************************************************************
9930 KQLEHLYDTLKQEFDAISREKHKLQEEVMKLKSMLRELQAARNQVSTVYTDLSGEETVES 180
WD   KQLEHLYDTLKQEFDAISREKHKLQEEVMKLKSMLRELQAARNQVSTVYTDLSGEETVES 180
GL3  KQLEHLYDTLKQEFDAISREKHKLQEEVMKLKSMLRELQAARNQVSTVYTDLSGEETVES 180
TMG1 KQLEHLYDTLKQEFDAISREKHKLQEEVMKLKSMLRELQAARNQVSTVYTDLSGEETVES 180
7088 KQLEHLYDTLKQEFDAISREKHKLQEEVMKLKSMLRELQAARNQVSTVYTDLSGEETVES 180
g38  KQLEHLYDTLKQEFDAISREKHKLQEEVMKLKSMLRELQAARNQVSTVYTDLSGEETVES 180
06-1 KQLEHLYDTLKQEFDAISREKHKLQEEVMKLKSMLRELQAARNQVSTVYTDLSGEETVES 180
422  KQLEHLYDTLKQEFDAISREKHKLQEEVMKLKSMLRELQAARNQVSTVYTDLSGEETVES 180
S05  KQLEHLYDTLKQEFDAISREKHKLQEEVMKLKSMLRELQAARNQVSTVYTDLSGEETVES 180
S06  KQLEHLYDTLKQEFDAISREKHKLQEEVMKLKSMLRELQAARNQVSTVYTDLSGEETVES 180
nps  KQLEHLYDTFKQEFDAISREKHKLQEEVMKLKSMLRELQAARNQVSTVYTDLSGEETVES 180
     *********:**************************************************
9930 TSVGAGCSSKPRPVAAAAAVAAANHYTTPPEQCNYVFNTEEYNPMSPAFWGSLPSSYHPQ 240
WD   TSVGAGCSSKPRPVAAAAAVAAANHYTTPPEQCNYVFNTEEYNPMSPAFWGSLPSSYHPQ 240
```

```
GL3  TSVGAGCSSKPRPVAAAAAVAAANHYTTPPEQCNYVFNTEEYNPMSPAFWGSLPSSYHPQ 240
TMG1 TSVGAGCSSKPRPVAAAAAVAAANHYTTPPEQCNYVFNTEEYNPMSPAFWGSLPSSYHPQ 240
7088 TSVGAGCSSKPRPVAAAAAVAAANHYTTPPEQCNYVFNTEEYNPMSPAFWGSLPSSYHPQ 240
g38  TSVGAGCSSKPRPVAAAAAVAAANHYTTPPEQCNYVFNTEEYNPMSPAFWGSLPSSYHPQ 240
06-1 TSVGAGCSSKPRPVAAAAAVAAANHYTTPPEQCNYVFNTEEYNPMSPAFWGSLPSSYHPQ 240
422 TSVGAGCSSKPRPVAAAAAVAAANHYTTPPEQCNYVFNTEEYNPMSPAFWGSLPSSYHPQ 240
S05 TSVGAGCSSKPRPVAAAAAVAAANHYTTPPEQCNYVFNTEEYNPMSPAFWGSLPSSYHPQ 240
S06 TSVGAGCSSKPRPVAAAAAVAAANHYTTPPEQCNYVFNTEEYNPMSPAFWGSLPSSYHPQ 240
nps TSVGAGCSSKPRPVAAAAAVAAANHYTTPPEQCNYVFNTEEYNPMSPAFWGSLPSSYHPQ 240
    ************************************************************
```
